# Chikungunya Virus in Febrile Humans and *Aedes aegypti* Mosquitoes, Yucatan, Mexico

**DOI:** 10.3201/eid2210.152087

**Published:** 2016-10

**Authors:** Nohemi Cigarroa-Toledo, Bradley J. Blitvich, Rosa C. Cetina-Trejo, Lourdes G. Talavera-Aguilar, Carlos M. Baak-Baak, Oswaldo M. Torres-Chablé, Md-Nafiz Hamid, Iddo Friedberg, Pedro González-Martinez, Gabriela Alonzo-Salomon, Elsy P. Rosado-Paredes, Nubia Rivero-Cárdenas, Guadalupe C. Reyes-Solis, Jose A. Farfan-Ale, Julian E. Garcia-Rejon, Carlos Machain-Williams

**Affiliations:** Universidad Autonoma de Yucatan, Merida, Mexico (N. Cigarroa-Toledo, R.C. Cetina-Trejo, L.G. Talavera-Aguilar, C.M. Baak-Baak, O.M. Torres-Chablé, P. González-Martinez, G. Alonzo-Salomon, E.P. Rosado-Paredes, N. Rivero-Cárdenas, G.C. Reyes-Solis, J.A. Farfan-Ale, J.E. Garcia-Rejon, C. Machain-Williams);; Iowa State University, Ames, Iowa, USA (B.J. Blitvich, M. Hamid, I. Friedberg)

**Keywords:** chikungunya, chikungunya virus, alphavirus, viruses, febrile humans, Aedes aegypti, mosquitoes, vector-borne infections, Yucatan, Mexico

## Abstract

Chikungunya virus (CHIKV) was isolated from 12 febrile humans in Yucatan, Mexico, in 2015. One patient was co-infected with dengue virus type 1. Two additional CHIKV isolates were obtained from *Aedes aegypti* mosquitoes collected in the homes of patients. Phylogenetic analysis showed that the CHIKV isolates belong to the Asian lineage.

Chikungunya virus (CHIKV; family *Togaviridae*, genus *Alphavirus*) is transmitted to humans by *Aedes* spp. mosquitoes (*1*,*2*). The virus is the etiologic agent of chikungunya, an acute febrile illness that is often accompanied by debilitating arthralgia. Historically, CHIKV has been restricted to the Eastern Hemisphere, but in 2013, the virus was reported in the Western Hemisphere during a large outbreak in the Caribbean region. CHIKV spread rapidly to South America, Central America, Mexico, and the United States. The Pan American Health Organization estimated that >1.7 million suspected and laboratory-confirmed cases of chikungunya have occurred in the Western Hemisphere (http://www.paho. org/hq/index.php?option=com_topics&view=readall&cid=5927&Itemid=40931&lang=en).

CHIKV was isolated in Mexico from a patient from Jalisco in whom symptoms developed in May 2014 shortly after the patient returned from the Caribbean region (*3*). The first autochthonous case was reported in October 2014 after CHIKV was isolated from a patient in southeastern state of Chiapas (*4*). CHIKV-infected *Aedes aegypti* mosquitoes and additional chikungunya cases were identified in Chiapas later in 2014 (*5*,*6*). To our knowledge, no reports of CHIKV in any other states in Mexico have been published. In this study, we tested febrile patients in the state of Yucatan and mosquitoes temporally and spatially associated with these patients for CHIKV infection.

## The Study

We obtained written informed consent from all patients who participated in the study or their legal guardians. The study population was composed of patients who came to hospitals or clinics in Yucatan during August–October 2015 with chikungunya-like illness. These patients were referred to the hematology laboratory at the Hideyo Noguchi Research Center (Merida, Yucatan, Mexico). A patient was considered to have chikungunya-like illness if he or she had fever and arthralgia. Travel history of each study participant was recorded, and any patient who had traveled outside Yucatan in the past 30 days before disease onset was excluded from the study.

Blood was collected from the cephalic vein of each patient, dispensed into a vacutainer tube (BD Diagnostics, Franklin Lakes, NJ, USA), and centrifuged. Serum was collected and stored at −80°C. Resting mosquitoes were collected from the homes of each study participant by using Centers for Disease Control and Prevention (Atlanta, GA, USA) backpack-mounted aspirators. Each house was examined once, and collections were made between 9:00 am and noon. All rooms were inspected, particularly dark areas (i.e., underneath furniture, in closets, and in curtains). Backyards were also searched, particularly shaded areas (i.e., pet homes, tool sheds, and underneath vegetation).

Mosquitoes were transported alive to the laboratory and identified on chill tables by using morphologic characteristics (*7*). Female mosquitoes were sorted into pools of <10 and homogenized in phosphate-buffered saline (pH 7.2) by using a mortar and pestle. Male mosquitoes were discarded.

An aliquot of each serum sample and mosquito homogenate was filtered and inoculated onto subconfluent monolayers of *Ae. albopictus* (C6/36) cells in 25-cm^2^ flasks. Cells were incubated for 7 days at 28°C. Second and third blind passages were performed in C6/36 and African green monkey kidney (Vero) cells, respectively. Vero cells were incubated for 3–7 days at 37°C in an atmosphere of 5% CO_2_. Cells were scraped from flasks after each passage and centrifuged at 10,000 × *g* for 10 min at 4°C. Supernatants were collected and stored at −80°C. Cell pellets were resuspended in Trizol (Invitrogen, Carlsbad, CA, USA), and total RNA was extracted following the manufacturer’s instructions.

We analyzed total RNA by using reverse transcription PCR (RT-PCR) and CHIKV-specific primers for a 107-nt region of the nonstructural protein 1 gene (primer sequences available upon request from the authors) and dengue virus (DENV)–specific primers for a 511-nt region of the capsid–membrane genes of all 4 serotypes (*8*). If DENV RNA was detected, we performed a semi-nested RT-PCR with serotype-specific primers. If CHIKV RNA was detected, we amplified a 3,744-nt region that spans the structural protein genes (capsid-E3-E2-6K-E1) (E, envelope; 6K, membrane-associated peptide) as 2 overlapping fragments (primer sequences available upon request from the authors).

Complementary DNAs were generated by using Superscript III reverse transcriptase (Invitrogen), and PCRs were performed by using *Taq* polymerase (Invitrogen). RT-PCR products were purified by using the Purelink Gel Extraction Kit (Invitrogen) and sequenced by using a 3730x1 DNA sequencer (Applied Biosystems, Foster City, CA, USA).

We isolated CHIKV from 12 (23.5%) of 51 study participants. DENV type 1 was also isolated from 1 CHIKV-positive patient. DENV was readily detected in cultured cells after the first blind passage, but its ability to replicate decreased after subsequent passages, presumably because CHIKV outcompeted this slower-replicating flavivirus.

The most common symptoms in patients infected with only CHIKV, in addition to fever, during the first 3 days of disease onset were arthralgia (100%), myalgia (100%), asthenia (90.9%), and rash (45.5%) (Table). Symptoms of the co-infected patient (a 31-year-old woman) included headache, myalgia, and rash. Age range of patients infected with only CHIKV was 9–59 years (mean age 31 years).

**Table:** Signs and symptoms of 12 patients infected with CHIKV during the first 3 days of disease onset, Yucatan, Mexico*

Sign/symptom	No. (%) patients	
	CHIKV infected, n = 11	Co-infected with DENV 1, n = 1
Arthralgia	11 (100.0)	1
Ankles	4 (36.4)	1
Knees	11 (100.0)	1
Shoulders	0 (0)	0
Wrists	11 (100.0)	1
Asthenia	10 (90.9)	0
Fever	11 (100.0)	1
Headache	0 (0)	1
Myalgia	11 (100.0)	1
Rash	5 (45.5)	1
Vomiting	1 (9.1)	0

*CHIKV, chikungunya virus; DENV 1, dengue virus type 1.

We collected a total of 237 female mosquitoes, and all were identified as *Ae. aegypti* mosquitoes. CHIKV was isolated from 2 pools. One pool contained mosquitoes collected in the living room of a 53-year-old patient who had a confirmed CHIKV infection. The other pool contained mosquitoes collected in bedroom of the co-infected patient. DENV was not isolated from any mosquitoes.

The capsid-E3-E2-6K-E1 region of each CHIKV isolate was sequenced and submitted to GenBank under accession nos. KU295117–KU295130. Pairwise alignments of the nucleotide and deduced amino acid sequences were performed by using Clustal Omega (http://www.ebi.ac.uk/Tools/ msa/clustalo/). Analysis showed that nucleotide sequences had 99.41%–99.97% identity and amino acid sequences 99.44%–100% identity with each other. The nucleotide sequence of 1 isolate (GenBank accession no. KU295121) was aligned with all other CHIKV sequences in GenBank and shown to have highest identity (99.52%) with the corresponding gene region of CHIKV isolates from Panama and El Salvador, followed by an identity of 99.49% with isolates from Chiapas, Mexico; Guatemala; Puerto Rico; Guyana; and elsewhere in the Western Hemisphere. Analysis of deduced amino acid sequences showed that mutations associated with increased infectivity of *Ae. albopictus* mosquitoes (E1-A226V and E2-L210Q) (*9*,*10*) were not present in genomes of any isolates.

Complete structural gene sequences of 60 CHIKV isolates, including the 14 isolates from Yucatan, were aligned by using MUSCLE (*11*), and phylogenetic trees were constructed by using the neighbor-joining algorithm as implemented in PHYLIP (*12*) (Figure). We observed 4 lineages, Asian, East/Central/South African, Indian Ocean, and West African lineages, which was consistent with results of previous studies (*1*,*5*). CHIKV isolates from Yucatan belonged to the Asian lineage and shared a close phylogenetic relationship with other isolates from the Western Hemisphere (Figure). Our isolates formed a nested clade within the Asian lineage. However, bootstrap support (0.61) for this topologic arrangement was not strong.

**Figure:**
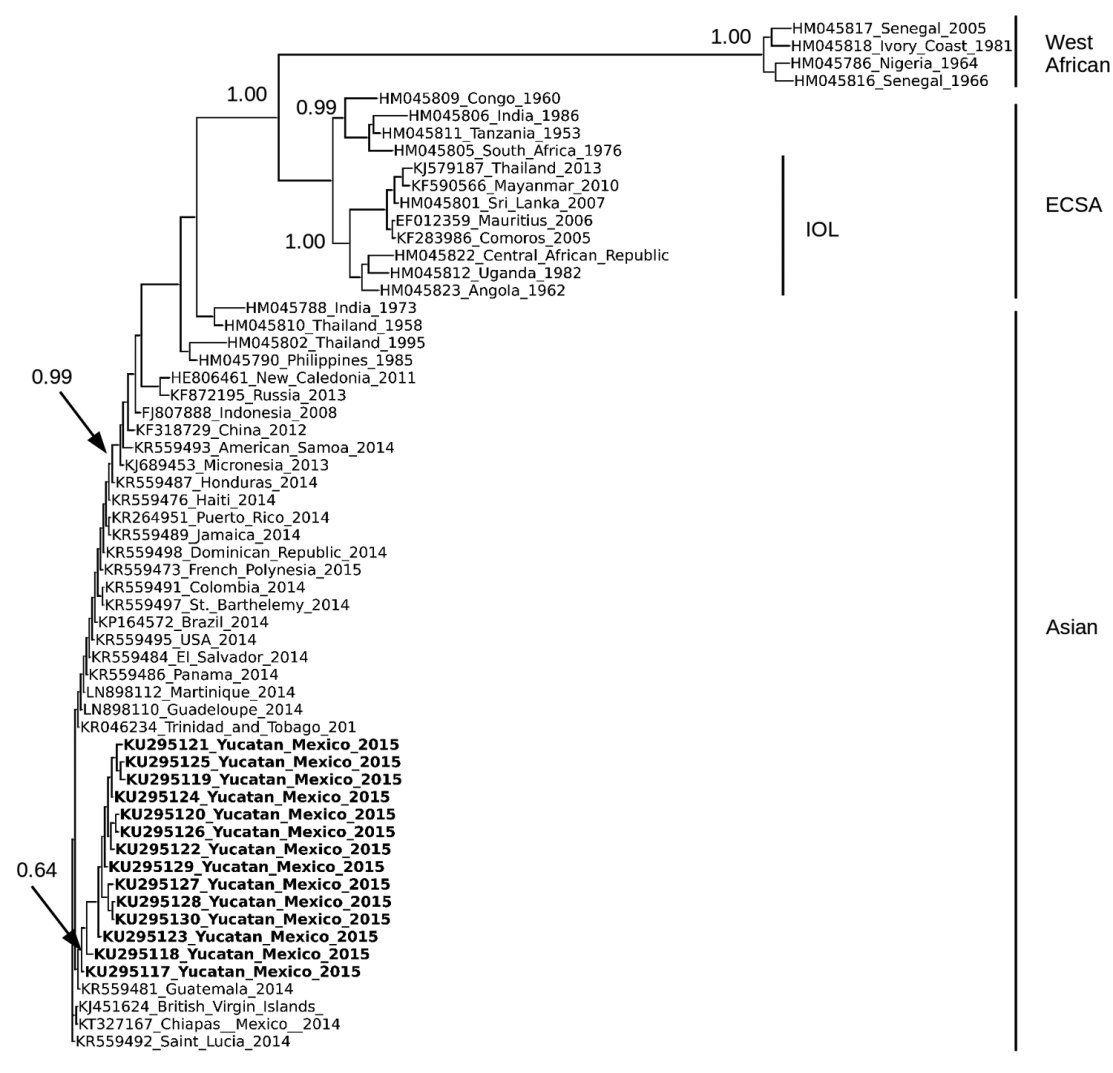
Phylogenetic analysis of chikungunya virus (CHIKV) isolates from Yucatan, Mexico. Analysis was based on a 3,744-nt structural gene region (capsid-E3-E2-6K-E1) of 63 CHIKV isolates, including the 14 isolates from Yucatan. Sequences were aligned by using MUSCLE (*11*), and the tree was constructed by using the neighbor-joining algorithm as implemented in PHYLIP (*12*) and using ETE3 (Environment for Tree Exploration 3) (*13*). Isolates are identified by GenBank accession number, country, and year isolated. CHIKV isolates from the Yucatan are shown in bold. Bootstrap values were generated by using 1,000 repetitions and normalized on a scale of 0–1. Bootstrap values for select branches are shown. 6K, membrane-associated peptide; E, envelope; ECSA, East/Central/South African lineage; IOL, Indian Ocean lineage.

## Conclusions

We isolated CHIKV from febrile patients and *Ae. aegypti* mosquitoes in Yucatan, Mexico, which provided additional evidence that this virus is spreading throughout the Americas at an alarming rate. Concurrent isolation of CHIKV and DENV from a patient in this study and patients in previous studies (*14**,**15*) underscores the need for differential diagnosis in areas where these viruses co-circulate.
